# Early childhood education and care (ECEC) during COVID‐19 boosts growth in language and executive function

**DOI:** 10.1002/icd.2241

**Published:** 2021-05-21

**Authors:** Catherine Davies, Alexandra Hendry, Shannon P. Gibson, Teodora Gliga, Michelle McGillion, Nayeli Gonzalez‐Gomez

**Affiliations:** ^1^ School of Languages, Cultures, and Societies University of Leeds Leeds UK; ^2^ Department of Experimental Psychology University of Oxford Oxford UK; ^3^ Department of Psychology Oxford Brookes University Oxford UK; ^4^ School of Psychology University of East Anglia Norwich UK; ^5^ Department of Psychology University of Warwick Coventry UK

**Keywords:** childcare, cognitive development, COVID‐19, executive functions, language development, socioeconomic status

## Abstract

High‐quality, centre‐based education and care during the early years benefit cognitive development, especially in children from disadvantaged backgrounds. During the COVID‐19 pandemic and its associated lockdowns, access to early childhood education and care (ECEC) was disrupted. We investigate how this period affected the developmental advantages typically offered by ECEC. Using parent‐report data from 189 families living in the UK, we explore associations between time spent in ECEC by 8‐to‐36‐month‐olds, their socioeconomic background, and their growth in language and executive functions between Spring and Winter 2020. Receptive vocabulary growth was greater in children who continued to attend ECEC during the period, with a stronger positive effect for children from less advantaged backgrounds. The growth of cognitive executive functions (CEFs) was boosted by ECEC attendance during the period, regardless of socioeconomic background. Our findings highlight the importance of high‐quality ECEC for the development of key skills and for levelling socioeconomic inequalities.

## 1 INTRODUCTION

High‐quality, centre‐based childcare during the first 3 years of life shows benefits for children's cognitive, language, and social development at school entry and beyond, across a range of national contexts (Barnes & Melhuish, 2017; Burchinal et al., 2000; Camilli, Vargas, Ryan, & Barnett, 2010; Drange & Havnes, 2019; Eryigit‐Madzwamuse & Barnes, 2014; Hansen & Hawkes, 2009; Melhuish et al., 2015; Melhuish & Gardiner, 2017; National Institute of Child Health and Human Development Early Child Care Research Network, 2000). These effects are particularly pronounced in children from disadvantaged backgrounds (Connell & Prinz, 2002; Felfe, Nollenberger, & Rodríguez‐Planas, 2015; Geoffroy et al., 2007, 2010; Larose, Côté, Ouellet‐Morin, Maughan, & Barker, 2020; Melhuish, 2004; Melhuish et al., 2015), meaning that investment in early childhood education and care (ECEC) is a powerful way of alleviating socioeconomic inequalities (Center on the Developing Child, 2010; Heckman, 2006).

A large body of work suggests that the amount that caregivers talk to their children varies as a function of socioeconomic background, with families who have to adapt to greater economic stress (e.g., lower income) and greater environmental stress (e.g., more income volatility; housing discrimination) providing less language input than their more advantaged peers (Ellwood‐Lowe, Foushee, & Srinivasan, 2020); for a review see (Schwab & Lew‐Williams, 2016)). A growing literature suggests that high‐quality ECEC plays a buffering role against these structural risk factors as well as against individual risk factors, for example, less consistent caregiving and less cognitively‐stimulating home environments (Côté, Borge, Geoffroy, Rutter, & Tremblay, 2008; Votruba‐Drzal, Levine Coley, & Lindsay Chase‐Lansdale, 2004). For example, household instability in early childhood predicted worse cognitive and social outcomes at age 5, but these associations were substantially reduced for children with access to ECEC (Berry et al., 2016). The protective effect of high‐quality ECEC stems from its provision of resources that may be lacking in the home environment. Within the childcare setting, space and facilities, structure and content of daily activities, staff turnover, and qualifications of care providers have been positively associated with children's cognitive outcomes (Hansen & Hawkes, 2009).

Here we investigate how links between ECEC, socioeconomic status (SES), and child development played out during the COVID‐19 pandemic. As the access to ECEC was severely restricted during this time, it is important to investigate the effects of closures on children. At the end of March 2020, ECEC settings in the UK were closed to all children except those of critical workers or those classed as vulnerable. Between March and June 2020, only 5–10% of children who usually attended ECEC did so (Department for Education [DfE], 2020). This was followed by an extended period of quarantine measures, reduced attendance, and disruption to ECEC (Bowyer‐Crane et al., 2020).

It is not yet known how the cognitive benefits of ECEC would be affected for those children who continued to attend settings during the lockdown. The attested benefits may be maintained for vulnerable children as they were encouraged to attend settings. Alternatively, benefits to this group may reduce due to the extensive disruption to ECEC staffing and facilities. It is also possible that the advantages usually enjoyed by children from higher‐SES backgrounds might reduce as families struggled to provide supportive learning environments at home while splitting their time between caring for young children, educating their other children, and working.

We explore these possible outcomes by analysing associations between ECEC attendance and measures of growth in two domains: language and Executive Functions (EFs; higher‐order skills which support the control of attention and behaviour in order to achieve goals). Both sets of skills have been shown to predict a range of cognitive, social, and academic outcomes (Bleses, Makransky, Dale, Højen, & Ari, 2016; Diamond, 2013; Roulstone, Law, Rush, Clegg, & Peters, 2011).

## 2 METHODS

### 2.1 Participants

Families with infants and children between 8 and 36 months of age from across the UK were recruited through online advertisements on research‐related websites and social media groups to take part in a study on language and cognitive development during the Covid 19 pandemic. Between March 23 and June 28, 2020 (henceforth “Spring 2020”) respondents answered questions relating to their socio‐demographic characteristics, use of formal (nursery, nanny, or childminder) and informal (family member) childcare, language(s) that their child was exposed to, their child's vocabulary development, EF‐related behaviours, birth factors (e.g., preterm birth), and several other factors not investigated here. Between November 27 and December 18, 2020 (henceforth “Winter 2020”), 6.5 months after the first observation (*M* days = 200, *SD* = 11.44), participants were asked to report again on their child's language ability and EF‐related behaviours, and several other factors not reported here. Only UK‐based infants under 36 months (*M* = 24.89, *SD* = 5.24) at the Winter 2020 data completion point, from monolingual English‐speaking families, with a gestational age of 37 weeks or over, and no known genetic conditions, are included in this study. One hundred and eighty‐nine eligible families completed this follow‐up questionnaire; only these participants are included in the current report. Ninety‐nine percent of respondents were the target child's mother, 1% their father. One hundred (53%) target children were male, 89 female. Vocabulary scores and a similar measure of ECEC attendance at the Spring observation point for most of our sample (*N* = 163) are also reported in Kartushina et al. (2021), which investigates separate questions on the impact of the home environment on language development.

This study received ethics approval from Oxford Brookes University Research Ethics Committee: ref 20023. All procedures performed in this manuscript were in accordance with the 1964 Helsinki Declaration and its later amendments or comparable ethical standards. All participating caregivers provided informed consent at each time‐point, on behalf of themselves and their child. On completion of the Spring 2020 questionnaires, families were given a £30 Amazon voucher. On completion of the Winter 2020 questionnaires, families were given a £5 Amazon voucher.

### 2.2 Socioeconomic status

Four indices of socioeconomic status (SES) were used in this study, as described below and summarized in Table 1.

1. Income: Parents were asked to report their total household income from one of the following categories: 1: £0–£20 k; 2: £21–£30 k; 3: £31–£40 k; 4: £41–£50 k; 5: £51–£60 k; 6: £61–£70 k; 7: £71 k or over.
2. Parental education: Parents were asked to report their highest level of education completed from one of the following categories: 1: Primary school; 2: Secondary school (this is the minimum legal requirement for formal education in the UK), 3: Sixth form or college: 4: Vocational college; 5: Undergraduate: 6: Postgraduate: 7: MBA; 8: Doctoral degree. For single/widowed parents, only their scores were used in the analyses; otherwise, mean scores were computed based on both parents.
3. An Index of Multiple Deprivation decile group was computed as a measure of neighbourhood deprivation from postcode data using either the English indices of deprivation (Noble et al., 2019), the Welsh Index of Multiple Deprivation (Welse Government, 2019), or the Scottish Index of Multiple Deprivation (Scottish Government, 2020) as appropriate.
4. Parents' occupational prestige: Parents were asked to report their occupation. This was converted into scores based on Hollingshead (1975) ranging from 1 to 9; whereby 1 is for cleaners or farm labourers, 5 is for clerical and sales workers, 7 is for owners of small businesses, managers, or journalists, and 9 is for executives, scientists, engineers, or large business owners. For single/widowed parents, only their scores were used in the analyses; otherwise, mean scores were computed based on all parents. If one parent was a full‐time homemaker, the occupation score was based on the other working parent.

**TABLE 1:** Demographic profile of participants

	Neighbourhood deprivation index	Household income	Parental education score	Parental occupation score
Mean (*SD*)	6.78 (2.50)	4.74 (1.96)	5.35 (1.27)	6.96 (1.58)

Ninety‐five percent of families were living in England, 4% in Scotland, and 1% in Wales. Prior to exploring the data, SES data were reduced to a single variable using Principal Components Analysis (PCA) on the full project sample (including bilingual families, not included here); see SM1. The extracted SES factor scores were used in the analyses reported below. The single measure aimed to capture the complex and multidimensional nature of SES (Navarro‐Carrillo, Alonso‐Ferres, Moya, & Valor‐Segura, 2020).

### 2.3 Language ability

The Oxford Communicative Development Inventory (O‐CDI) (Hamilton, Plunkett, & Schafer, 2000) was used to assess children's vocabulary development. This UK measure uses the parental report to assess comprehension and production of 416 early English words across 19 different categories (e.g., animals, vehicles, food, and drink). Parents of children aged 18 to 36 months completed the extended version of the O‐CDI which includes 133 additional items (i.e., 549 English words) and four additional categories (i.e., online, adventures, parts of things, and parts of animals). Parents were instructed to mark each word that they thought their child “understood” (receptive vocabulary) or “understood and said” (expressive vocabulary). The variables of interest were children's raw receptive and expressive scores.

### 2.4 Executive functions

Parent‐report of emergent EFs was collected using the Early Executive Functions Questionnaire (EEFQ) (Hendry & Holmboe, 2020). The EEFQ comprises 31 items relating to the control of attention, behaviour, and emotion; see https://osf.io/fa5eq for details. Parents are asked to report on a 7‐item Likert scale how often their child has exhibited a particular behaviour during the preceding fortnight (28 items) – or, for behaviours that may be uncommon in all children, or highly context‐dependent, play a short game with their child designed to elicit a particular skill and then report back on their child's performance (3 items). After conducting Confirmatory Factor Analysis to confirm the factor structure identified by Hendry and Holmboe (2020), and to establish measurement invariance by age (see SM1.2), we computed a Cognitive Executive Function (CEF) score by calculating the mean of all items targeting inhibitory control, working memory, and cognitive flexibility. We also computed a separate Regulation score from the mean of all Regulation items. Internal consistency was excellent for the CEF composite and Regulation scales at both Spring 2020 and Winter 2020 observation points; see Table 2. CEF and Regulation scores were computed only where a minimum of 70% applicable items were complete; see Table 3 for final sample size.

**TABLE 2:** Internal consistency (Cronbach's alpha) of EEFQ CEF factor and Regulation scale

	CEF	Regulation
Spring 2020	.875	.876
Winter 2020	.829	.886

**TABLE 3:** ECEC access by SES group (median split)

	Lower SES	Higher SES
ECEC prior to Spring Lockdown (days per week)	.78 (1.08)	1.69 (1.72)
ECEC during 2020 pandemic	.51 (.69)	1.28 (1.16)

*Note*: Cells show mean scores with *SDs* in parentheses.

CEF and Regulation scores were not significantly associated at the Spring 2020 (*r* = −.011, *p* = .884) or Winter 2020 observations (*r* = .138, *p* = .060). CEF scores showed high homotypic stability between Spring and Winter 2020 (*r* = .746, *p* < .001), as did Regulation scores (*r* = .612, *p* < .001).

### 2.5 Early childhood education and care

Parents were asked whether their child received non‐parental childcare from a nursery, childcare setting, or nanny – henceforth ECEC – before and during the Spring Lockdown, between lockdowns, and again during the Winter lockdown, and if so, to report the duration (full or half days), frequency (days per week), date resumed (if discontinued due to the Spring Lockdown) and degree of disruption (weeks prevented from accessing ECEC due to, for example, staff shortages, quarantining of close contacts); see SM2 for the full measure. From this information, we computed the total number of days the child accessed ECEC, and then subtracted the number of disrupted days to compute a total score that was then divided by the number of weeks elapsed since the start of the Spring Lockdown to compute an ECEC score (mean number of days per week; see Table 3). ECEC data were available for all except 1 participant, who indicated in the free text that they used a nursery but did not provide quantitative data and therefore were excluded from analyses.

In addition, at the Winter lockdown, parents were asked whether their child received non‐parental childcare from a member of the extended family (e.g., grandparents, aunt, uncle) – henceforth Informal Childcare – and if so, to report the duration (full or half days) and frequency (days per week) this was used. From this information, we computed the total number of days the child accessed informal childcare, which was then divided by the number of weeks elapsed since the start of the Spring Lockdown to compute an InformalChildcare score (mean number of days per week). Note that it was assumed that no informal childcare was accessed during the Spring Lockdown, due to the nature of the restrictions at the time. Informal Childcare data were available for all except 1 participant, as above.

### 2.6 Statistical analysis

We were interested both in absolute change in language and EF skills over the pandemic period, and in changes in skills relative to age expectations. To compute absolute change, raw Receptive Vocabulary, Expressive Vocabulary, CEF, and Regulation scores at Spring 2020 were subtracted from raw scores from the same measure at Winter 2020 to produce a simple difference score (diffReceptive, diffExpressive, diffCEF, diffRegulation). To compute changes in skills above and beyond age‐related change, raw Receptive Vocabulary, Expressive Vocabulary, CEF, and Regulation scores at each timepoint were regressed on age at that time point, to produce a Spring 2020 and Winter 2020 age‐controlled score for each measure; see SM1.3. We then computed a Latent Change Score (LCS) for each measure (LCSReceptive, LCSExpressive, LCSCEF, LCSRegulation) with the age‐controlled score as independent variables, using a script derived from Kievit et al. (2017). LCS has an advantage over simple difference scores whilst retaining the value of a repeated measure by separating the variables into structural “error‐free” latent components and measurement error, using the principles of Structural Equation Modelling (SEM) but requiring only a minimum of 2‐time points. The latent component represents the “true” change between adjacent time points (McArdle, 2009). For this study, as LCS scores were computed using age‐controlled scores, a positive score means that in Winter 2020 the child is now further ahead for their age than they were at Spring 2020, whilst a negative score means that the child has progressed more slowly rather than indicating a frank loss of skills. As shown in SM3, we used an MLR estimator, enabling us to compute LCS scores for the 3 participants with missing Winter 2020 CEF scores and the 1 participant with missing Winter 2020 Regulation scores.

Multiple linear regression analyses were then conducted using difference scores for each of the language and EF measures as dependent variables. Predictor variables were standardized ECEC, SES, and age at the Winter 2020 observation point, and interaction terms for ECEC with SES, and ECEC with age, computed by multiplying the standardized variables. These continuous independent variables were entered as simultaneous predictors in the regression models. For comparison purposes, we also conducted equivalent multiple linear regression analyses using Informal Childcare instead of ECEC.

To aid with interpretation, we present plots showing the regression of language and EF difference scores on ECEC. Data are grouped into higher and lower SES, using a median split group to illustrate possible interactions with SES. In addition, we present plots showing the regression of language and EF LCS scores on ECEC, by SES group, to aid with interpretation of associations between ECEC and growth in language and EF, after accounting for age.

## 3 RESULTS

Summary descriptive data are presented in Table 4.

**TABLE 4:** Descriptive data for participants

	Mean	*SD*	Min	Max	*N*
Age at Spring 2020 (months)	18.30	5.23	8.09	29.33	189
Age at Winter 2020 (months)	24.39	5.26	14.00	35.00	189
ECEC (days per week)	.90	1.03	0.00	4.86	188
InformalChildcare (days per week)	.21	.55	0.00	3.79	188
SES	.01	1.02	−2.55	2.01	189
Receptive vocabulary: Spring 2020	199.48	153.40	0	533	189
Receptive vocabulary: Winter 2020	408.10	121.25	56	549	189
diffReceptive	208.62	93.30	12	442	189
LCSReceptive	.00	42.94	−157.70	11.70	189
Expressive vocabulary: Spring 2020	85.32	124.85	0	509	189
Expressive vocabulary: Winter 2020	299.82	184.36	0	549	189
diffExpressive	214.50	140.54	0	526	189
LCSExpressive	.00	99.58	−219.69	250.06	189
CEF: Spring 2020	4.57	.74	2.27	6.30	189
CEF: Winter 2020	4.92	.64	2.71	6.05	186
diffCEF	.35	.51	−1.20	1.83	186
LCSCEF	.00	0.23	−0.76	0.68	189
Regulation: Spring 2020	5.32	1.01	2.00	7.00	189
Regulation: Winter 2020	5.23	1.02	2.13	6.88	188
diffRegulation	−.09	.89	−3.62	3.50	188
LCSRegulation	5.23	.54	3.30	6.72	189

Abbreviations: CEF, Cognitive Executive Function; ECEC, Early Childhood Education and Care; LCS, Latent Change Score.

### 3.1 Effects of age, ECEC, and SES on language skills

**FIGURE 1:**
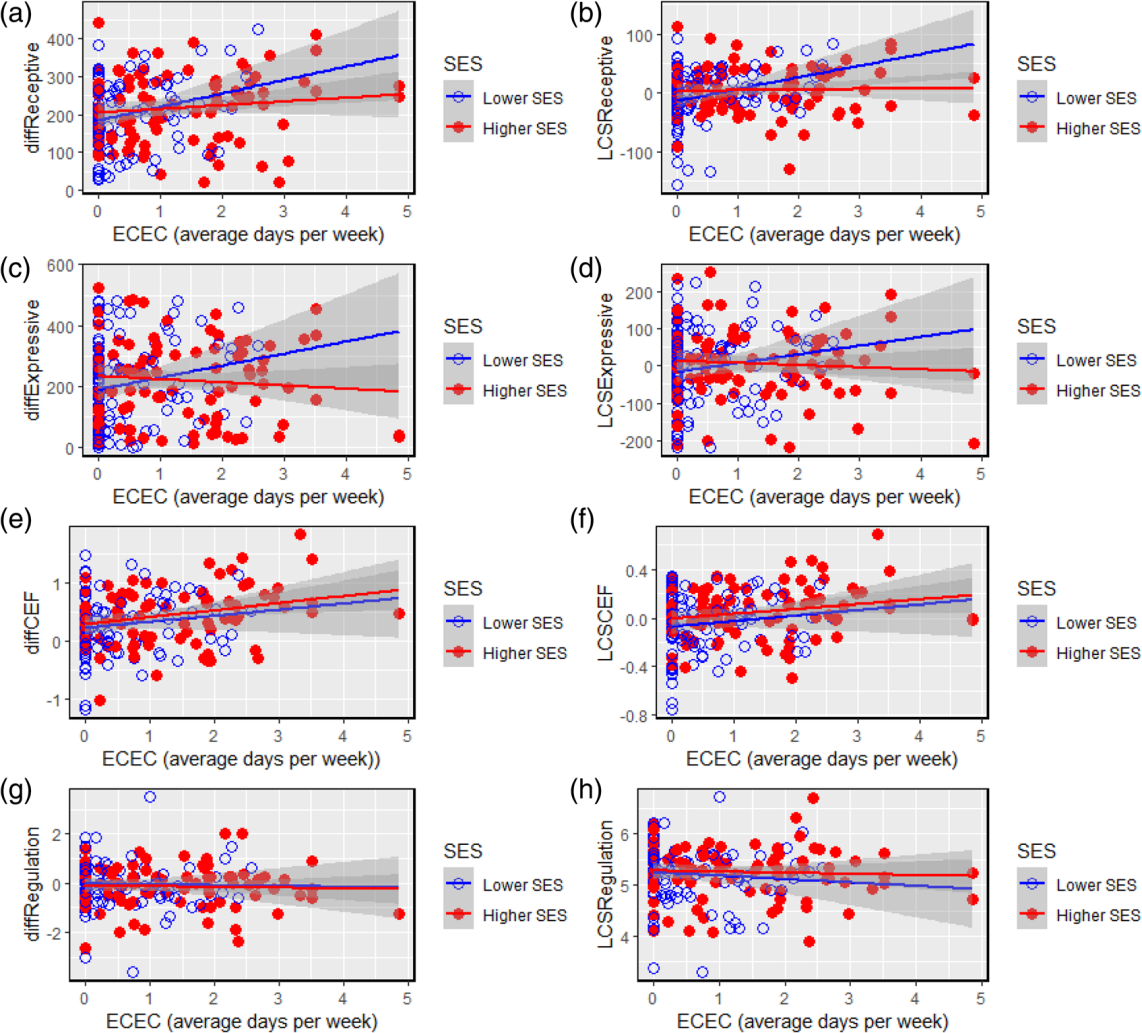
Associations between Early Childhood Education (ECEC) and changes in language (a–d) and EF skills (e–h) during the 2020 pandemic, by SES group (median split). Raw difference scores are used for figures a,c,e,g. Latent change in age‐controlled scores are presented to aid interpretation in figures b,d,f,h

As shown in Table 5, increases in receptive vocabulary between Spring and Winter 2020 (“diffReceptive”) were negatively associated with age, such that growth in language skills was more pronounced for younger children compared with older children. Increases in receptive vocabulary were also positively associated with ECEC, such that more exposure to ECEC during the 2020 pandemic was associated with greater increases in receptive vocabulary during that period: beta = 23.55, meaning that a child who regularly accessed 1 day of ECEC per week during the pandemic could be expected to understand 24 more new words over the Spring–Winter 2020 period compared with their peers, whilst a child who regularly accessed 2 days of ECEC per week during the pandemic could be expected to understand 48 more new words over the Spring–Winter 2020 period compared with their peers. There was no significant interaction between age and ECEC; that is, the benefits of ECEC on receptive vocabulary were no more pronounced for younger children than for older children (or vice versa). There was no significant main effect of SES, but there was a significant interaction between SES and ECEC, such that the benefits of ECEC on receptive vocabulary were more pronounced for children from lower‐SES backgrounds (see Figure 1a). Figure 1b, using LCSs computed with age‐controlled data illustrates this data in an alternative way; children from lower‐SES backgrounds showed greater increases in receptive vocabulary for their age the more ECEC they received, whereas ECEC exposure was not significantly associated with increases in receptive vocabulary after accounting for age for children from higher‐SES backgrounds.

**TABLE 5:** Multiple linear regressions of growth in language and EF scores on ECEC and SES, using raw difference scores between Spring and Winter 2020

	diffReceptive	diffExpressive	diffCEF	diffRegulation
Predictor	Β	β	β	β
Age	−.446***	.325**	−.295***	.064
ECEC	.255**	.062	.255**	−.075
SES	.022	.102	.019	.062
ECEC‐Age interaction	−.066	−.008	.029	.007
ECEC‐SES interaction	−.194*	−.152	−.052	.003
Adjusted *R* ^2^	.247	.120	.131	−.018

Abbreviations: β, Standardized beta; SES, Socioeconomic status.

*Note*:***p < .001, **p < .01, *p < .05.

**FIGURE 2:**
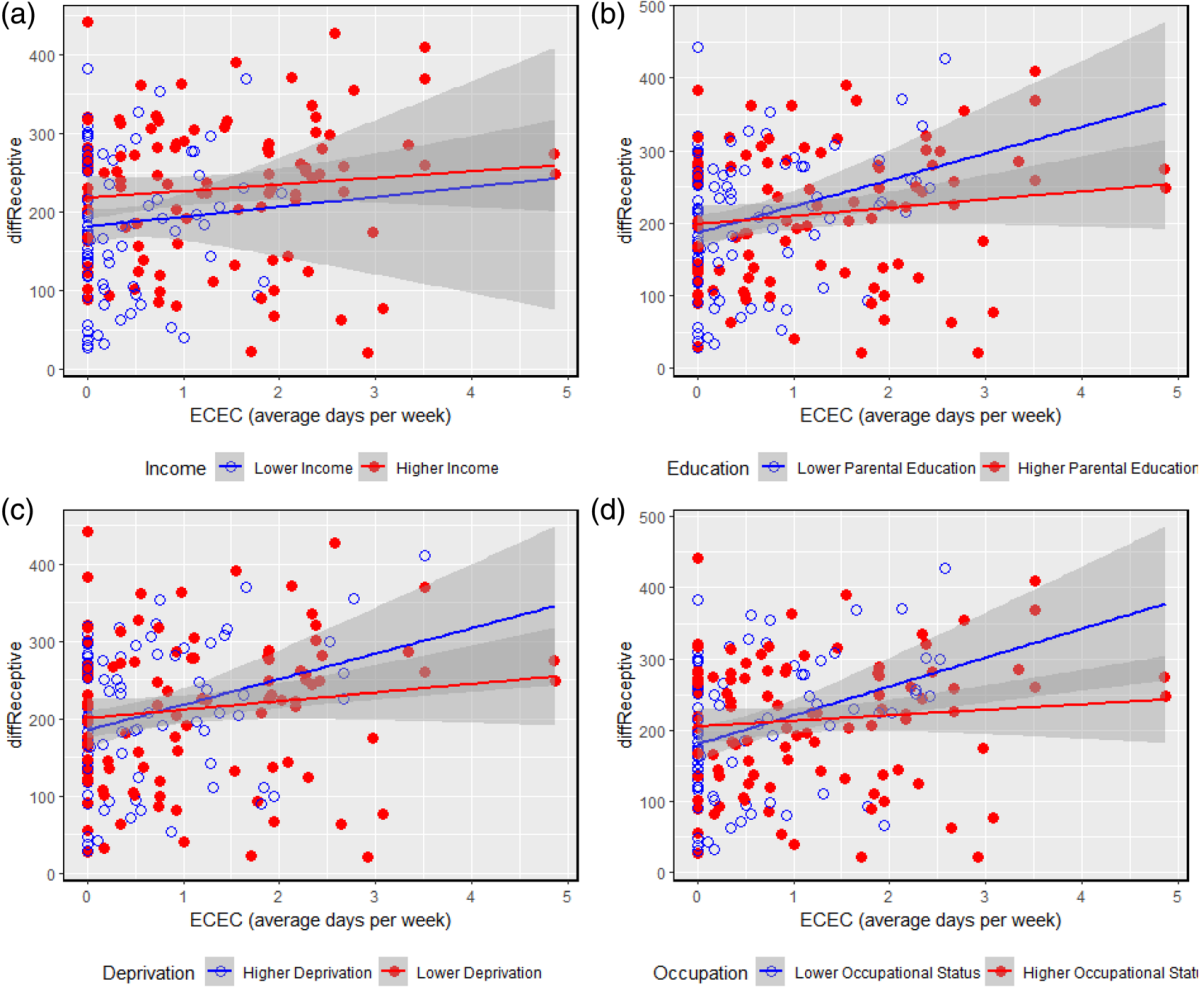
Associations between Early Childhood Education (ECEC) and changes in receptive language skills during the 2020 pandemic, by Parental Income (a), Parental Education (b), Neighbourhood Deprivation (c), and Parental Occupational Status (d) (median split used for each index)

On the request of a reviewer, results were further explored at the level of each specific SES metric to better understand whether ECEC‐outcome associations were influenced by any aspects of SES in particular. As shown in Table 6, when individual indicators of SES were used, the interaction term with ECEC and SES indicator was significantly associated with Receptive Language difference scores only for the Parental Income and Parental Occupational Status indicators. Nevertheless, as shown in Figure 2, there was also a consistent trend for the benefits of ECEC to be more pronounced for infants from lower‐SES backgrounds, when Parental Education and Neighbourhood Deprivation were used as the SES index.

**TABLE 6:** Multiple linear regressions of growth in language and EF scores on ECEC and individual indicators of SES (in bold), using raw difference scores between Spring and Winter 2020

	diffReceptive	diffExpressive	diffCEF	diffRegulation
Predictor	β	β	β	β
Age	−.444***	.330**	−.292***	.068
ECEC	.216*	.116	.214*	−.042
**Parental income**	.022	−.003	.096	.018
ECEC‐Age interaction	−.077	−.005	.021	.012
ECEC‐ **Parental income** interaction	−.164*	−.163	−.042	−.027
Adjusted *R* ^2^	.244	.106	.140	−.019

Predictor	β	β	β	β
Age	−.444***	.316**	−.291***	.065
ECEC	.186*	.012	.263**	−.057
**Parental education**	−.018	.135	−.035	.013
ECEC‐Age interaction	−.068	−.011	.031	.006
ECEC‐ **Parental education** interaction	−.075	−.066	−.017	.022
Adjusted *R* ^2^	.223	.107	.129	−.020

Predictor	β	β	β	β
Age	−.455***	.325**	−.290***	.066
ECEC	.209**	.068	.214**	−.042
**Neighbourhood deprivation**	.033	.036	.072	−.015
ECEC‐Age interaction	−.102	−.027	.031	.012
ECEC‐ **Neighbourhood deprivation** interaction	−.125	−.095	.031	.006
Adjusted *R* ^2^	.231	.094	.132	−.020

Predictor	β	β	β	β
Age	−.446***	.323	−.290***	.059
ECEC	.187**	.041	.276***	−.077
**Parental occupation**	−.006	.103	−.056	.115
ECEC‐Age interaction	−.069	−.007	.038	.006
ECEC‐ **Parental occupation** interaction	−.151*	−.135	−.090	−.012
Adjusted *R* ^2^	.241	.119	.136	−.007

Abbreviations: β, Standardized beta; SES, Socioeconomic status.

*Note*: ***p < .001, **p < .01, *p < .05.

We considered whether families in which one parent stayed at home to provide full‐time childcare may be more likely to have high occupational status scores (for these families only the employed parent's occupational status was used), and whether this may have influenced our results. When only families in which both parents worked outside the home were included (*n* = 162), the interaction between ECEC and Parental Occupational Status was in the same direction as for the whole sample, but was no longer a significant predictor of Receptive Language growth (*β* = −.126, *p* = .086,). Yet, the interaction between ECEC and SES (i.e., the PCA‐derived score) was still a significant predictor of Receptive Language growth (*β* = −.200, *p* = .019) when only families in which both parents worked outside the home were included. We, therefore, conclude that the particular benefits of ECEC on the receptive language skills of children from lower‐SES families are best interpreted when viewing SES as a multidimensional construct that includes the interaction of cultural and economic factors.

As shown in Table 5, increases in expressive vocabulary between Spring and Winter 2020 (“diffExpressive”) were positively associated with age, such that older children showed greater increases compared with younger children. There was no significant main effect of SES or ECEC on expressive vocabulary increases. The interaction between SES and childcare did not reach significance thresholds, but as shown in Figure 1c,d was in a consistent direction with that of receptive vocabulary whereby benefits of ECEC on expressive vocabulary appear more pronounced for children from lower‐SES backgrounds. Consistent results were found when extreme ECEC scores (more than 2 *SD* above or below the mean) were excluded; see Table S1.4 and Figure S1 in Data S1. As shown in Table 6, no new significant interactions with ECEC were found for Expressive Language when individual indicators of SES were used.

As shown in Table 7, there was no effect of informal childcare, nor any effect of interactions between informal childcare and SES or age, on either receptive or expressive vocabulary.

**TABLE 7:** Multiple linear regressions of growth in language and EF scores on informal childcare and SES, using raw difference scores between Spring and Winter 2020

	diffReceptive	diffExpressive	diffCEF	diffRegulation
Predictor	β	β	β	Β
Age	−.447***	.320**	−.308***	.083
InformalChildcare	−.112	.003	.021	.070
SES	.079	.134	.148	.050
InformalChildcare ‐Age interaction	.097	.002	.026	−.094
InformalChildcare ‐SES interaction	.048	.075	.006	−.021
Adjusted *R* ^2^	.230	.109	.091	−.005

Abbreviations: β, Standardized beta; SES, Socioeconomic status.

*Note*: ***p < .001, **p < .01, *p < .05.

### 3.2 Effects of age, ECEC and SES on EF skills

As shown in Table 5, increases in CEF between Spring and Winter 2020 (“diffCEF”) were negatively associated with age, such that such that growth in executive function skills was more pronounced for younger children compared with older children. Increases in CEF were also positively associated with ECEC, such that more exposure to ECEC during the 2020 pandemic was associated with greater increases in CEF during that period: beta = 0.135, such that a child who regularly accessed 5 days of ECEC per week during the pandemic would be expected to have increased their CEF score by .68 between Spring and Winter 2020, in comparison to the group average increase of .35 across the same period. There was no significant interaction between age and ECEC; that is, the benefits of ECEC on CEF were no more pronounced for younger children than for older children (or vice versa).

There was no significant main effect of SES, and no significant interaction between SES and ECEC; see also Figure 1e. Figure 1f, using LCSs computed with age‐controlled data illustrates this data in an alternative way; the more ECEC children received, the greater increases in CEF for their age they showed, regardless of SES background. Consistent results were found when extreme ECEC scores (more than 2 *SD* above or below the mean) were excluded; see Table S1.4 and Figure S1 in Data S1. As shown in Table 6, no new significant interactions with ECEC were found to predict CEF when individual indicators of SES were used.

As shown in Tables 5 and 6 and Figure 1g,h, changes in regulation scores between Spring and Winter 2020 were not significantly related to age, ECEC, or SES over the pandemic period.

As shown in Table 7, there was no effect of informal childcare, nor any effect of interactions between informal childcare and SES or age, on either CEF or Regulation.

## 4 DISCUSSION

This exploratory study examined associations between two aspects of young children's environment: time spent in ECEC and their SES, and growth in aspects of their cognitive development: expressive and receptive vocabulary, CEFs, and regulation, measured during the 2020 COVID‐19 crisis in a cohort of families living in the UK.

We aimed to analyse the effects of restricted access to ECEC during the pandemic. This is crucial for informing policy in the event of further lockdowns, and when planning measures to remediate the impacts of ECEC disruptions. Our exploration of how the typical advantages of ECEC were affected by the lockdown revealed differential effects between the two outcome measures. Lower‐SES children who continued to attend ECEC showed enhanced language benefits. This suggests that children from less affluent backgrounds who lost access were disproportionately disadvantaged by the social distancing measures. There was no effect of SES on the ECEC‐linked growth in CEFs.

Our data showing that ECEC during the pandemic boosted the growth of receptive vocabulary in children from less advantaged backgrounds align with previous work from non‐pandemic times that finds similar benefits of ECEC on the language abilities of disadvantaged children (Berry et al., 2016; Drange & Havnes, 2019; Geoffroy et al., 2007, 2010; Larose et al., 2020).

The selective effect for lower‐SES children may be due to ECEC's enrichment of the language input at home (Vernon‐Feagans, Bratsch‐Hines, & Investigators, 2013). Children from socioeconomically disadvantaged backgrounds tend to have more limited language skills (Locke, Ginsborg, & Peers, 2002), a difference which may emerge from as early as 18 months (Fernald, Marchman, & Weisleder, 2013). Although there are many reasons for the link between social disadvantage and language ability, evidence suggests that family background is associated with aspects of language input important in development, such as the amount of speech that children hear, lexical diversity, and conversational turn‐taking, as well as parental responsiveness, degree of directing behaviour, and incorporation of language goals in play (Hammer & Weiss, 1999; Hart & Risley, 1995; Hirsh‐Pasek et al., 2015; Hoff, 2003a, 2003b; Nicely, Tamis‐LeMonda, & Bornstein, 1999; Rowe, 2012); see Schwab and Lew‐Williams (2016) for a review. In situations where input quality is more limited, the impact of ECEC practitioners' interactions is likely to be greater. This protective effect is likely to be stronger still during the pandemic. Lower‐income families have been disproportionately impacted by an increased prevalence of infections, deaths, unemployment, and mental ill‐health (Kousoulis et al., 2020; Office for National Statistics [ONS], 2021; Shum et al., 2021), all stressors which are likely to negatively affect home interactions with children.

Our data show that ECEC attendance was positively associated with CEFs, with no significant interaction effect of SES and ECEC: that is, ECEC appears to boost the growth of young children's emerging CEFs, regardless of their socioeconomic background. The benefits of ECEC on EF development may be due to ECEC's provision of developmentally appropriate learning materials and adult‐child interactions which scaffold learning, and have been shown to promote child EFs (Amso, Salhi, & Badre, 2019; Clark et al., 2013; DeJoseph, Sifre, Raver, Blair, & Berry, 2021; Rosen et al., 2020). In pre‐pandemic contexts, access to these EF‐promoting factors in the home is greater for children of parents with higher‐SES (Amso et al., 2019; DeJoseph et al., 2021; Devine, Bignardi, & Hughes, 2016; Rosen et al., 2020). Recent research indicates that, overall, engagement in enriching activities was not higher for more advantaged families during the 2020 pandemic (Hendry et al., in prep), which might explain why ECEC‐benefits extend across the socioeconomic spectrum.

Although our findings contrast with US‐based reports that childcare hours are weakly negatively‐associated with preschoolers' EFs (Son & Chang, 2018), none in our sample exceeded an average of 4.9 days per week, such that potential negative effects of excessive ECEC use would not have been detected. Son and Chang (2018) found that quality of childcare positively predicted preschoolers' EFs: as our measure of ECEC was time, rather than quality‐based, it may be the case that highly‐structured ECEC delivered through fewer contact hours would offer greater benefits for early EFs than our data show. We found no effect of age, ECEC, or SES on the growth of regulation, consistent with previous reports (Son & Chang, 2018).

Interestingly, informal childcare did not yield the same benefits on either language or CEF growth. Although the evidence is mixed regarding the cognitive benefits of informal care (Green, Pearce, Parkes, Robertson, & Katikireddi, 2020; Hansen & Hawkes, 2009; Laing & Bergelson, 2019; Melhuish et al., 2015), ECEC's strengths in terms of, for example, caregiver‐child interactions, predictable schedules, lower screen use, and caregiver education is likely to be important for nurturing children from disadvantaged contexts (Dowsett, Huston, Imes, & Gennetian, 2008). Note that we did not collect fine‐grained information about the nature of this informal childcare at the first observation point since restrictions did not allow household mixing. However, some families might have accessed such care, for example, through intergenerational living. Future work should investigate the relative effects of childcare type during the pandemic, integrating the extent of the disruption to ECEC conditions.

Our findings yield several policy recommendations. Our data have highlighted the clear benefits of ECEC on children's cognitive development, and the disproportionate penalty for less advantaged children who lost access to ECEC. We recommend that settings remain open for vulnerable children throughout future lockdowns – with appropriate protection for staff – as a means of alleviating inequalities. Further, we propose that vulnerable children who missed out in 2020 are prioritized for extra funded hours in the following years.

More broadly, there are ongoing concerns about the low take‐up of funded places for 2‐year‐olds in England, where nationally 68% of the eligible 2‐year‐olds benefit from funded ECEC (Department for Education [DfE], 2019), with a significantly lower take‐up in certain areas. This low take‐up by less advantaged families is also evident in our sample (see Table 5). We recommend that funded places are promoted in target areas, and administrative barriers to their take‐up removed.

The study has two main limitations. First, despite its efficiency for data collection while social distancing, our use of parent‐report increases the likelihood of error and recall bias. Relatedly, as our ECEC measure probed quantity but not quality, we are limited in our conclusions about exactly how ECEC confers developmental advantages. Future studies should therefore involve collaboration with the ECEC sector, and include questions about for example, activities, facilities, and practitioner qualifications. Second, we used a self‐selecting convenience sample of UK parents, presenting limits on generalisability. We also had relatively low representation from families with extremely low SES, skewing the sample towards highly‐educated parents (who were more likely to use ECEC).

We have demonstrated that as early as infancy, ECEC boosts cognitive development, that is, vocabulary (lower‐SES in particular) and CEFs (all children). Solid skills in these areas are likely to have cascading positive effects as children move through their preschool years and beyond. To maintain these benefits for child development and for levelling inequalities, properly‐funded, high‐quality ECEC is crucial.

## CONFLICT OF INTEREST

The authors report no conflicts of interest.

## ETHICS STATEMENT

This study received ethics approval from the Oxford Brookes University Research Ethics Committee (UREC).

## Supporting information


**Data S1.** Supporting information.

**Data S2.** Supporting information.

## Data Availability

The data that support the findings of this study are available from the corresponding author upon reasonable request.
