# Supplementary material for: Early childhood education and care (ECEC) during COVID‐19 boosts growth in language and executive function
Source: Infant Child Dev. 2021 May 21;30(4):e2241. doi: 10.1002/icd.2241 (PMC8236989; doi:10.1002/icd.2241)
Supplement: Supplementary file 1 — Data S1. Supporting information. [file ICD-30-0-s002.docx]

Supplementary Materials 1

# 1.1 Socio-Economic Status

To reduce the number of comparisons required, Principal Components Analysis (PCA) was conducted on the imputed demographic dataset for all participants recruited to the project (including multilingual families, those with Spring 2020 data only, and those with demographic data only; *n*=892). Only one PCA factor had an eigenvalue over Kaiser’s criterion of 1. This factor, which we labelled SES, explained 55% of the variance. The extracted SES factor scores were used in the analyses reported in the main manuscript.

*Supplementary Table 1.1.* PCA factor loadings

|  | SES factor loadings |
| --- | --- |
| Parental Occupation Score | .828 |
| Household Income | .823 |
| Parental Education Score | .781 |
| Neighbourhood deprivation index | .528 |

# 1.2 Confirmatory Factor Analysis of EEFQ data

In order to establish whether the EEFQ data showed the same structure as in Hendry and Holmboe (2020), we conducted Confirmatory Factor Analysis (CFA) on the items previously found to map to the Cognitive Executive Function (CEF) factor, using the Winter 2020 data.

CFA was conducted in RStudio v1.2.5033 using the lavaan package vn 0.6-7 (Rosseel, 2012). The ML estimator was used to deal with missing data. The unitary CEF model established in Hendry and Holmboe (2020), showed good model fit in our data; see Supplementary Table 1.2.

To investigate the impact of age on the measurement model, we compared model fit for infants younger versus older than 30 months. This split was chosen as measurement invariance by age has previously been established for infants under 30 months (Hendry and Holmboe, 2020). As shown in Supplementary Table 1.2, the unitary CEF model (Model 1) showed poor model fit when configural invariance for age group was assumed. After reviewing factor loadings, the Working Memory game was dropped from the model as it performed poorly in the youngest age group. The revised model showed adequate configural invariance; see Supplementary Table 1.2.

There was a significant difference in model fit between the configural invariance model and the weak factorial invariance model (diff CFI = -.037). When factor loadings for 3 items (IC3R, WM2R and IC game; see Hendry and Holmboe, 2020 for item mappings) were allowed to vary by group, the difference in model fit between the configural invariance model and the weak factorial invariance model was no longer significant (diff CFI =-.007). There was a significant difference in model fit between the weak invariance model and a model in which intercepts and factor loadings (except the 3 items listed above) were held equivalent for the same items discussed above across age bands (diff CFI = -.076). When intercepts for 7 items (IC1-3R, FX2R, FX5, IC game and FX game) were allowed to vary between age groups, CFI indices were comparable (diff CFI =-.009), thus meeting criteria for partial strong factorial invariance in terms of age. Factor scores computed allowing for partial strong factorial invariance were highly correlated with raw composite scores (r=.910, *p*<.001), therefore raw scores were used in subsequent analysis for ease of interpretability and to enable future comparisons with other datasets.

*Supplementary Table 1.2* Tests of measurement invariance (Winter 2020 data – up to 36 months)

| Model | RMSEA [90% CI] | SRMR | CFI |
| --- | --- | --- | --- |
| 1 Unitary CEF model | .063 [.045, .081] | .043 | .956 |
| 1a Configural invariance | .063 [.043, .082] | .072 | .862 |
| 1b Configural invariance – no WM game | .087 [.061, .111] | .061 | .946 |
| 1b Weak factorial invariance | .097 [.076, .117] | .080 | .909 |
| 1b Weak partial factorial invariance (loadings for 3 items allowed to vary) | .081 [.058, .103] | .076 | .939 |
| 1b Strong factorial invariance | .108 [.090, .125] | .095 | .863 |
| 1c Partial strong factorial invariance (loadings for 3 items and intercept for X items allowed to vary) | .080 [.058, .100] | .080 | .930 |

# 1.3 Age-controlled Language and EEFQ scores

Regression analyses were conducted to identify the effects of age on Receptive and Expressive vocabulary, and CEF and Regulation scores at both time-points. As shown in Supplementary Table 1.3, there was a linear effect of age for Spring 2020 Receptive vocabulary; a quadratic effect of age for Winter 2020 Receptive vocabulary, Spring and Winter 2020 Expressive vocabulary, Spring and Winter 2020 CEF, and Spring 2020 Regulation. Residuals from these analyses were saved for use in the LCS analyses as described in the main manuscript. For Winter 2020 Regulation, there was no effect of age, therefore the raw Regulation scores were used in the LCS analyses.

*Table SM1.3* Regression analyses of language and EF scores on age

|  |  | *Spring 2020* | | | | | | | | | | |
| --- | --- | --- | --- | --- | --- | --- | --- | --- | --- | --- | --- | --- |
|  |  | **Receptive vocabulary** | | **Expressive vocabulary** | | | **CEF** | | | **Regulation** | | |
| Model | Age term | β | Adj R^2^ | β | Adj R^2^ | β | | Adj R^2^ | β | | Adj R^2^ |  |
| 1 | Linear | .864*** | .746 | .737*** | .541 | .568*** | | .319 | -.175* | | .025 |  |
| 2 | Linear | .706** | .745 | -.809* | .600 | 1.555*** | | .337 | -1.484** | | .059 |  |
|  | Quadratic | .160 |  | 1.566*** |  | -.998* | |  | 1.323** | |  |  |
|  |  | *Winter 2020* | | | | | | | | | | |
|  |  | **Receptive vocabulary** | | **Expressive vocabulary** | | | **CEF** | | | **Regulation** | | |
| Model | Age term | β | Adj R^2^ | β | Adj R^2^ | β | | Adj R^2^ | β | | Adj R^2^ |  |
| 1 | Linear | .727*** | .526 | .740 | .545 | .422*** | | .174 | -.110 | | .007 |  |
| 2 | Linear | 2.951*** | .584 | 2.163*** | .568 | 1.664** | | .189 | -.371 | | .002 |  |
|  | Quadratic | -2.237*** |  | -1.431** |  | -1.249* | |  | .262 | |  |  |

β: Standardized beta
****p*<.001, ***p*<.01, **p*<.05

# 1.4 Analyses excluding extreme ECEC values

*Supplementary Table 1.4*. Multiple linear regressions of growth in language and EF scores on ECEC (excluding extreme values) and SES, using raw difference scores between Spring and Winter 2020.

|  | diffReceptive | diffExpressive | diffCEF | diffRegulation |
| --- | --- | --- | --- | --- |
| Predictor | β | β | β | β |
| Age | -.443*** | .332** | -.325*** | .057 |
| ECEC | .196** | .051 | .182* | -.042 |
| SES | -.012 | .118 | .016 | .062 |
| ECEC-Age interaction | -.065 | -.009 | -.041 | -.028 |
| ECEC-SES interaction | -.203** | -.125 | -.083 | .023 |
| Adjusted *R*^2^ | .237 | .125 | .120 | -.022 |

β: Standardized beta. SES: Socioeconomic status.

****p*<.001, ***p*<.01, **p*<.05


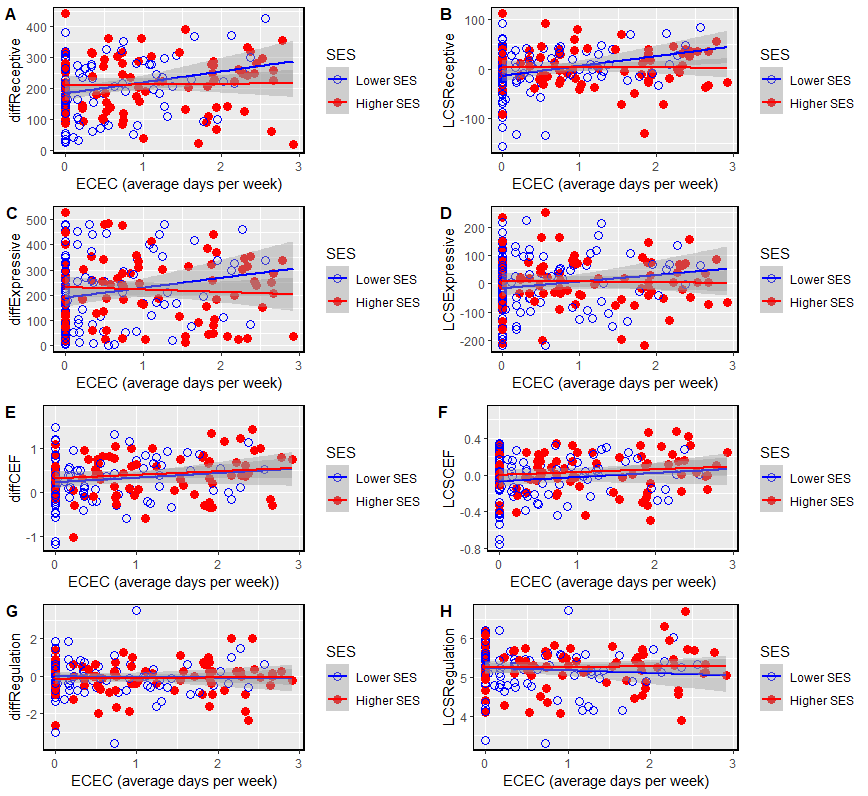


*Supplementary Figure 1.4.* Associations between Early Childhood Education (ECEC) with extreme values excluded, and changes in language (A-D) and EF skills (E-H) during the 2020 pandemic, by SES group (median split). Raw difference scores are used for figures A, C, E and G. Latent change in age-controlled scores are presented to aid interpretation in figures B, D, F and H.
