# Supplementary material for: Early childhood education and care (ECEC) during COVID‐19 boosts growth in language and executive function
Source: Infant Child Dev. 2021 May 21;30(4):e2241. doi: 10.1002/icd.2241 (PMC8236989; doi:10.1002/icd.2241)
Supplement: Supplementary file 2 — Data S2. Supporting information. [file ICD-30-0-s001.docx]

# Pre-Lockdown and Spring Lockdown Childcare Questionnaire

What kind of childcare does your child usually have?

- Parents (1)
- Nursery (2)
- Childminder (3)
- Other: (4) ________________________________________________

Was your child attending nursery before the quarantine/social distancing measures started?

- Yes (1)
- No (2)

*Display This Question:*

*If Was your child attending nursery before the quarantine/social distancing measures started? = No*

Was your 8-to-36-month-old child attending a childminder before the quarantine/social distancing measures started?

- Yes (1)
- No (2)

*Display This Question:*

*If Was your child attending nursery before the quarantine/social distancing measures started? = Yes*

*Or Was your 8-to-36-month-old child attending a childminder before the quarantine/social distancing... = Yes*

How many months has your child been going to childcare?

________________________________________________________________

*Display This Question:*

*If Was your child attending nursery before the quarantine/social distancing measures started? = Yes*

*Or Was your 8-to-36-month-old child attending a childminder before the quarantine/social distancing... = Yes*

| 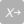 |
| --- |

Days How many days per week?

▼ 1 (1) ... 7 (7)

*Display This Question:*

*If Was your child attending nursery before the quarantine/social distancing measures started? = Yes*

*Or Was your 8-to-36-month-old child attending a childminder before the quarantine/social distancing... = Yes*

On average, did your child attend childcare...

- Full-time (1)
- Half time (2)

Are you considered a key worker by the government?

- Yes (1)
- No (2)

Has your child attended childcare since the social distancing measures started in March?

- Yes (1)
- No (2)

*Display This Question:*

*If Has your child attended childcare since the social distancing measures started in March? = Yes*

| 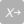 |
| --- |

Since the social distancing measures started, how many days per week?

▼ 1 (1) ... 7 (7)

*Display This Question:*

*If Has your child attended childcare since the social distancing measures started in March? = Yes*

Since the social distancing measures started, on average, did your child attend childcare...

- Full-time (1)
- Half time (2)

*Display This Question:*

*If Has your child attended childcare since the social distancing measures started in March? = No*

Has your child started attending childcare since the lockdown eased?

- Yes (1)
- No (2)

*Display This Question:*

*If Has your child started attending childcare since the lockdown eased? = Yes*

| 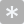 |
| --- |

When did your child start attending childcare again?

________________________________________________________________

# Winter Lockdown Childcare Questionnaire

Was your child going to nursery or a childminder before the Spring Lockdown/social distancing measures started in March 2020?

- Yes (1)
- No (2)

*Display This Question:*

*If Was your child going to nursery or a childminder before the Spring Lockdown/social distancing mea... = Yes*

Did your child return to their usual nursery or childminder after the Spring Lockdown was lifted (June 2020)

- Yes (1)
- No (2)
- My child continued to attend their nursery or childminder during lockdown (4)

*Display This Question:*

*If Did your child return to their usual nursery or childminder after the Spring Lockdown was lifted... = Yes*

| 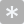 |
| --- |

When did your child return to their usual nursery or childminder after the Spring Lockdown was lifted?

________________________________________________________________

*Display This Question:*

*If Did your child return to their usual nursery or childminder after the Spring Lockdown was lifted... = No*

*Or Was your child going to nursery or a childminder before the Spring Lockdown/social distancing mea... = No*

What childcare arrangements did you make after the Spring Lockdown was lifted (June 2020)? Please select as many as apply.

- My child was looked after by their usual caregiver(s) (e.g. parents) at home (1)
- My child was looked after by a member of the extended family (e.g. grandparents, aunt, uncle) (2)
- My child attended a new nursery or childcare setting (3)
- My child was looked after by a nanny at home (4)
- Other, please specify: (5) ________________________________________________

*Display This Question:*

*If What childcare arrangements did you make after the Spring Lockdown was lifted (June 2020)? Please... = My child attended a new nursery or childcare setting*

| 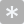 |
| --- |

When did your child start attending their nursery/childcare setting after the Spring Lockdown was lifted?

________________________________________________________________

*Display This Question:*

*If Did your child return to their usual nursery or childminder after the Spring Lockdown was lifted... = Yes*

*Or Did your child return to their usual nursery or childminder after the Spring Lockdown was lifted... = My child continued to attend their nursery or childminder during lockdown*

*Or What childcare arrangements did you make after the Spring Lockdown was lifted (June 2020)? Please... = My child attended a new nursery or childcare setting*

On average, how many days per week does your child attend their nursery/childcare setting?

▼ 1 (1) ... 7 (7)

*Display This Question:*

*If Did your child return to their usual nursery or childminder after the Spring Lockdown was lifted... = Yes*

*Or Did your child return to their usual nursery or childminder after the Spring Lockdown was lifted... = My child continued to attend their nursery or childminder during lockdown*

*Or What childcare arrangements did you make after the Spring Lockdown was lifted (June 2020)? Please... = My child attended a new nursery or childcare setting*

On average, did your child attend childcare...

- Full-time (1)
- Half-time (2)

*Display This Question:*

*If Did your child return to their usual nursery or childminder after the Spring Lockdown was lifted... = Yes*

*Or Did your child return to their usual nursery or childminder after the Spring Lockdown was lifted... = My child continued to attend their nursery or childminder during lockdown*

Has your child been prevented from attending their nursery/childcare setting since their return due to nursery-imposed COVID measures, e.g. staff shortages, bubbles quarantining?

- Yes (1)
- No (2)

*Display This Question:*

*If What childcare arrangements did you make after the Spring Lockdown was lifted (June 2020)? Please... = My child attended a new nursery or childcare setting*

Has your child been prevented from attending their new nursery/childcare setting since starting due to nursery-imposed COVID measures, e.g. staff shortages, bubbles quarantining?

- Yes (1)
- No (2)

*Display This Question:*

*If Has your child been prevented from attending their nursery/childcare setting since their return d... = Yes*

*Or Has your child been prevented from attending their new nursery/childcare setting since starting d... = Yes*

*Or Did your child return to their usual nursery or childminder after the Spring Lockdown was lifted... = My child continued to attend their nursery or childminder during lockdown*

How many weeks between June and December has your child been prevented from attending their nursery/childcare setting? For example, their setting closed due to staff shortages or bubbles quarantining

▼ Less than 1 week (8) ... More than 6 weeks (7)

| Page Break |  |
| --- | --- |

*Display This Question:*

*If What childcare arrangements did you make after the Spring Lockdown was lifted (June 2020)? Please... = My child was looked after by a member of the extended family (e.g. grandparents, aunt, uncle)*

On average, how many days per week is your child looked after by a member of the extended family (e.g. grandparents, aunt, uncle)

▼ 1 (1) ... 7 (7)

*Display This Question:*

*If What childcare arrangements did you make after the Spring Lockdown was lifted (June 2020)? Please... = My child was looked after by a member of the extended family (e.g. grandparents, aunt, uncle)*

On average, is your child looked after by a member of the extended family (e.g. grandparents, aunt, uncle) for...

- Full-day (1)
- Half-day (2)

*Display This Question:*

*If What childcare arrangements did you make after the Spring Lockdown was lifted (June 2020)? Please... = My child was looked after by a nanny at home*

On average, how many days per week is your child looked after by a nanny at home?

▼ 1 (1) ... 7 (7)

*Display This Question:*

*If What childcare arrangements did you make after the Spring Lockdown was lifted (June 2020)? Please... = My child was looked after by a nanny at home*

On average, is your child looked after by a nanny at home for...

- Full-day (1)
- Half-day (2)
